# An audiometric study of the effects of paraspinal stimulation on hearing acuity in human subjects – understanding the Harvey Lillard phenomenon

**DOI:** 10.1186/s12998-014-0039-2

**Published:** 2014-11-19

**Authors:** Mark Demers, Zehra Gajic, Everett Gerretsen, Brian Budgell

**Affiliations:** Canadian Memorial Chiropractic College, 6100 Leslie St., Toronto, ON M2H 3 J1 Canada

**Keywords:** (3–10) Audiometry, Hearing, Cochlear nucleus, Somatic stimulation, Muscle, Human

## Abstract

**Background:**

The founder of chiropractic, Daniel David Palmer, constructed a model of causation of disease based on his seminal experience with a patient, Harvey Lillard, who lost his hearing at the instant of injuring his upper back, but had his hearing restored suddenly 17 years later after receiving spinal manipulation. Palmer’s model of disease causation, that of displaced vertebrae impinging on spinal nerves and thereby disrupting the innervation of dependent organs, was in fact incongruent with what was known at the time about human neuroanatomy and neurophysiology. The current study proposes and tests an alternative hypothesis: that increased afferent input from paraspinal muscles attenuates the central transmission of auditory information.

**Methods:**

Between September 13 and November 13, 2013, forty healthy young adults were recruited and randomly divided into two cohorts: one receiving successive trials of sham TENS, and the second receiving sham and then authentic TENS. During the administration of sham and authentic TENS to the upper thoracic spine, hearing acuity was measured to determine perception thresholds at the frequencies normally tested clinically.

**Results:**

In the first cohort, there were no differences in perception thresholds in the first and second trials of sham TENS, speaking to the reliability of the testing process. In the second cohort, there were no significant differences in perception thresholds during sham and authentic TENS.

**Conclusions:**

Within the constraints of the current study design, including demographic characteristics and TENS parameters, there was no evidence that innocuous afferent input to upper thoracic paraspinal muscles modulated thresholds of audibility.

## Background

The founder of modern chiropractic, Daniel David Palmer, attributed his discovery of the underlying mechanisms of spinal manipulation to his clinical experience with two patients. The first of these was Harvey Lillard, a gentleman who reported sudden deafness coincident with an acute strain to his upper back. Palmer found a thoracic vertebra ‘racked’ from its normal position and reasoned that if he replaced the vertebra by spinal manipulation, the patient’s hearing would be restored [1]. As it turned out, he was correct in his clinical prediction. Palmer subsequently encountered a patient with some sort of cardiac problem and, coincidently, ‘displaced’ thoracic vertebrae. He reasoned that the displaced vertebrae were somehow impinging on the sympathetic nerves to the heart and thereby impairing cardiac function. He reported that restoration of the vertebrae to their normal position was accompanied by resolution of the patient’s cardiac complaint. Palmer went on to explain:

*Then I began to reason if two diseases, so dissimilar as deafness and heart trouble, came from impingement, a pressure on nerves, were not other disease due to a similar cause? Thus the science (knowledge) and art (adjusting) of Chiropractic were formed at that time. I then began a systematic investigation for the cause of all diseases and have been amply rewarded.*

Palmer’s theory of the causation of disease was grounded in what was then current understanding of the anatomy of the sympathetic nervous system. It was recognized that sympathetic nerves arising from each segment of the thoracolumbar spinal cord projected to (and, it was assumed, regulated) specific adjacent visceral organs. It was therefore reasonable to conclude that impingement of any particular spinal nerve might lead to dysfunction in a segmentally-linked organ. By way of example, a subluxation at the T4 level might well cause cardiac disease, whereas a subluxation at the T7 or T8 level might well cause disease affecting the stomach. In fact, this model of the causation of disease had been articulated earlier by others [2], but Palmer elevated it to a unifying cause of virtually all disease. This model had great appeal among practitioners of the day, and seemed to vindicate clinical reports of localized spinal injuries being associated with disorders of specific viscera.

Today, the segmental sympathetic innervation of the thoracic and abdominal viscera is an established concept. We understand that there may be some variation from individual to individual, and that there may be some overlap of nerve territories – any given visceral organ may receive sympathetic innervation from several adjacent spinal segments, and any given spinal segment may project to several different organs [3]. Nonetheless, in broad strokes ‘impingement’ (whatever that might be) of a particular spinal nerve could well account for some kind of visceral dysfunction – except for the case of Harvey Lillard. There is no ‘thoraco-cochlear nerve’ to be impinged; only the most circuitous peripheral connections between the upper thoracic spine and the organs of hearing. And so, we are at something of a loss to explain the Harvey Lillard phenomenon.

It would be easy enough to dismiss the case of Harvey Lillard if there were not a number of other reports of similar cases – patients losing their hearing as a result of a spinal injury, or having their hearing restored by spinal manipulation; see, for example [4,5]. Thus, the phenomenon itself seems real enough; what is lacking is a plausible mechanism.

As an alternative to Palmer’s theory of nerve impingement, it has been suggested that the altered sensory input from Harvey Lillard’s back injury may have altered the central processing of information from his ears [6]. In more detail, information from the inner ear travels to the cochlear nucleus and from there is transmitted to the cerebral cortex, which allows for the conscious experience of hearing. The cochlear nucleus also receives sensory input from muscles of vocalization, eating, and vigorous respiration. This somatic sensory information inhibits activity of auditory cells in the cochlear nucleus, dampening hearing acuity. The muscle groups listed all generate sounds internally when active. This means that hearing acuity is decreased when there are internally generated sounds and increased when there are only externally generated sounds. It has been proposed that this mechanism is advantageous as it enhances the contrast of sounds, allowing for awareness of surroundings, ultimately increasing chances of survival [7].

The dampening of hearing due to increased afferent input from axial muscles (of vocalization, eating and respiration) is well documented in animals; see, for example [8], but has not been tested in humans. Hence, the purpose of this study was to determine whether increased afferent input from upper thoracic paraspinal muscles reduces hearing acuity in otherwise healthy young humans.

## Methods

### Study setting

This study was conducted between September 13 and November 13, 2013 within the media services recording studio of Canadian Memorial Chiropractic College, Toronto, Canada.

### Subjects

The study sample consisted of 40 healthy third year chiropractic students (19 male, 21 female) with a mean age of 25 years (range 22–36), verbally recruited from a class of 182 students. None of the subjects had a history of hearing complaints. On the day of testing, none of the subjects had neck or upper back pain which disrupted or caused them to modify their daily activities. During the week prior to testing, none of the subjects received or self-administered any treatment for neck or upper back pain.

### Procedures

Using a random number generator, subjects were randomly divided into control and treatment cohorts of 19 and 21 subjects, respectively. Each of the cohorts received an initial period of sham transcutaneous electrical nerve stimulation (TENS) to the upper back. Stimulating electrodes (Blue Sensor, Ambu A/S, Denmark) were placed approximately 2 cm on either side of the T4-T5 junction. The electrodes were connected to a computer driven stimulus isolator (ML180, ADInstruments, Boulder, CO) out of the line of sight of the subject. Subjects received a train of 500 μsec square wave stimuli at 100Hz, with the current increased incrementally until they indicated that they could perceive some sensation at the electrode contacts. The current was incrementally reduced until the subjects indicated that they could no longer detect it. Then, the current was turned off, but subjects were led to believe that it was continuing at a low level. Thus, an attempt was made to deceive subjects into thinking they were receiving authentic TENS, regardless of their actual assignment. In this state, hearing acuity in the left and right ears was measured as described below.

Subsequently, the control cohort received a second period of sham TENS and the treatment cohort received a period of authentic TENS. Authentic TENS was delivered at 100Hz, 500 μsec square waves at an intensity determined by the subject to just initiate muscle contraction (mean ± SD = 6.2 ± 2.4 mA, range 2.9 to 10.0 mA). All participants in the authentic TENS group denied discomfort; one subject in the control cohort reported discomfort and asked for the level of current to be reduced. At no time during the testing process were the subjects told what treatment they were receiving.

During the two periods of treatment, each participant had their hearing thresholds measured with an audiometer (Pocket-Audiometer, v2.0) running on an iPhone G3 (Apple Inc, Cupertino, CA) at a conventional range of frequencies: 125, 250, 500, 1000, 2000, 4000 and 8000 Hz. Tones were presented in a random order, and subjects were asked to signal by hand when they detected a tone. Tones were maintained for up to 5 seconds for detection, and each threshold was confirmed by a second trial. The tones were delivered through noise-cancelling headphones (Model MDR-NC7, Sony Electronics Inc, San Diego, CA), to reduce the influence of extraneous noises. The procedure was conducted in a quiet recording studio, rather than a sound-proof booth, an environment which is likely adequate to detect even small changes in perception threshold using noise-cancelling headphones [9,10]. All thresholds were recorded by one investigator who did not participate in data analysis and who was blinded to the records of previous trials.

### Statistical analysis

Hearing thresholds for each tone frequency (mean ± standard deviation) were measured separately for the left and right ears using paired t-tests for comparisons between values obtained with sham TENS and i) a second trial of sham TENS or ii) a trial of authentic TENS. Additionally, for the control cohort, Cronbach’s alpha was calculated for the repetition of each measure (subject × ear × frequency) of perception threshold.

### Ethics approval

This study was approved by the Research Ethics Board of Canadian Memorial Chiropractic College (approval #1308X07).

## Results

All recruited subjects completed the study and no data sets were discarded. Table 1 shows the mean hearing thresholds for the control cohort which received 2 successive trials of sham TENS. There were small and statistically insignificant or no changes in mean thresholds between the first and second exposures to sham TENS. Cronbach’s alpha for the repetition of all measures (ear x frequency) was 0.96 indicating an extremely high rate of repeatability.

**Table 1:** **Mean hearing thresholds – sham TENS cohort**

	**Threshold - Mean (S.D.) decibels**					
	**Left Ear**			**Right Ear**		
**Freq (Hz)**	**Sham TENS trial 1**	**Sham TENS trial 2**	**p value**	**Sham TENS trial 1**	**Sham TENS trial 2**	**p value**
**125**	6.8 (3.8)	6.3 (3.7)	0.67	7.6 (3.9)	5.8 (1.9)	0.07
**250**	11.1 (4.6)	10.8 (3.4)	0.84	10 (0)	9.7 (1.1)	0.32
**500**	20.5 (5.2)	19.2 (4.5)	0.41	19.2 (3.4)	18.9 (2.7)	0.79
**1000**	16.8 (7.1)	16.3 (7.4)	0.82	14.7 (2.6)	15.3 (2.0)	0.49
**2000**	18.2 (3.8)	17.4 (3.9)	0.53	15.8 (3.0)	16.1 (2.1)	0.76
**4000**	19.5 (7.6)	17.1 (7.5)	0.34	18.7 (5.5)	17.6 (5.9)	0.57
**8000**	10.3 (8.1)	9.5 (8.5)	0.77	9.5 (8.2)	10.5 (8.2)	0.69

Control cohort (i.e. 2 successive treatments of sham TENS); mean (S.D.) thresholds for the cohort at each frequency are expressed in decibels for the left and right ears.

Table 2 shows the mean hearing thresholds for the treatment cohort which received 1 trial of sham TENS followed by a trial of authentic TENS. There were small and statistically insignificant or no changes in mean thresholds between the first and second trials, with one exception. Only for the testing of the 1000 Hz frequency in the left ear was there a p value of 0.04, and within the context of the multiple comparisons conducted in this study, this would conventionally be regarded as not statistically significant.

**Table 2:** **Mean hearing thresholds – authentic TENS cohort**

	**Threshold - Mean (S.D.) decibels**					
	**Left Ear**			**Right Ear**		
**Freq (Hz)**	**Sham TENS trial 1**	**Authentic TENS trial 2**	**p value**	**Sham TENS trial 1**	**Authentic TENS trial 2**	**p value**
**125**	6.9 (2.5)	6.9 (2.5)	1	7.1 (2.5)	6.9 (2.5)	0.76
**250**	10 (0)	10 (0)	1	10 (0)	10 (0)	1
**500**	20 (2.7)	19.0 (2.6)	0.25	19.5 (3.5)	19.3 (3.3)	0.82
**1000**	15.7 (1.8)	14.8 (1.1)	0.04	16.4 (2.3)	15.5 (2.2)	0.18
**2000**	18.3 (2.9)	17.4 (3.0)	0.30	18.1 (3.7)	17.4 (3.8)	0.54
**4000**	20.2 (8.9)	18.8 (8.4)	0.59	17.9 (4.4)	18.3 (4.8)	0.74
**8000**	11.4 (7.1)	10.2 (6.2)	0.57	8.1 (3.7)	8.3 (2.9)	0.82

Authentic cohort (i.e. trial 1 of sham TENS followed by trial 2 of authentic TENS); mean (S.D.) thresholds for the cohort at each frequency are expressed in decibels for the left and right ears.

Further analysis of Table 2 results (Sham TENS vs Authentic TENS) yields effect sizes (mean difference divided by standard deviation of differences) for the effect of authentic TENS on hearing acuity ranging from 0.47 in the direction of a negative effect (TENS decreases perception threshold/increases hearing acuity) to 0.18 in the direction of a positive effect (TENS increases perception threshold/decreases hearing acuity). Based on the largest observed effect size in the positive direction (0.18) the required sample size to conclude statistical significance at the 0.05 level with a power of 0.8, we would require a replication study with approximately 250 subjects. This effect size, however, was the largest among all observed effects – a strategy sometimes referred in the vernacular as ‘cherry picking.’ Had we chosen to work with effect sizes for any of the other sets of comparisons, sizes of required cohorts for replication studies would have been greater. Further, these thresholds of statistical significance were for changes in hearing acuity which were likely to be clinically trivial.

At the conclusion of the second trial, it was revealed to subjects that they may have been assigned to receive either sham or authentic TENS. On questioning, all of the subjects who received authentic TENS correctly guessed that they had been assigned to the authentic TENS cohort. Further, 10 of 19 subjects in the control cohort correctly guessed that they had received a sham treatment, while 9 of 19 subjects believed that they had received authentic TENS.

## Discussion

This study sought to determine whether innocuous somatic stimulation, in the form of TENS, applied to the T4/T5 paraspinal tissues modulated the perception thresholds for frequencies commonly tested clinically. The study was designed to test a hypothesis concerning the mechanism underlying the Harvey Lillard phenomenon – hearing loss associated with an upper back injury. Hence, stimulation was applied at what is thought to be the approximate site of Harvey Lillard’s injury, and a stimulation modality was chosen to target tissues which were likely involved, at least to some degree, in Mr. Lillard’s case. In fact, the procedure failed to produce evidence that innocuous stimulation, which included stimulation of T4/T5 paraspinal muscles, had any effect on hearing acuity in our test cohort.

Repeated trials of sham TENS revealed no changes in perception thresholds on average and a high repeatability for individual measures (subject × ear × frequency). This argues against a learning effect whereby the subjects might become better able to detect tones with repeated exposures. Similarly, it argues against test fatigue. Thus the testing procedure itself appears to have a good level of intra-examiner reliability. Consequently, if TENS had appeared to have produced any effect, it would like have been specifically due to the stimulation. However, the challenge in this instance is accounting for the lack of effect. One possible explanation is that although there is a real effect of TENS on hearing acuity, the number of subjects in this study was inadequate – the study was ‘under-powered.’ As detailed above, a power calculation revealed that even for the largest positive results, the effect size was so small that replication with statistically significant results would require approximately 250 subjects. Furthermore, in most instances, the small treatment effects which were achieved were in the opposite direction from that proposed by our test hypothesis. On this basis, we conclude that our hypothesis of TENS (at the stimulation parameters used in this study) dampening hearing acuity to be convincingly disproven.

This does not argue against cross-modal interactions, for example between hearing and somatosensory stimulation, as have been documented in a number of species; see for example [11]. In humans, such interactions likely have an adaptive advantage in speech recognition, learning and articulation [12]. In animals, the ability to block out self-generated sounds may aid in the detection of externally generated sounds from, for example, prey or predators [7,13].

The lack of effect of TENS in this instance may be due to several factors such as the precise placement of electrodes which thereby determines which muscles are preferentially stimulated. Alternative sites of stimulation might produce different results. Additionally, the nature of the stimulation may have been inappropriate. It has recently been reported that in an animal model TENS applied to the paws at parameters regarded as noxious suppressed the activity of neurons in the medial geniculate body which receive auditory information from the cochlear nucleus [14]. Donishi et al. argued that this might have an adaptive advantage in focusing the subject’s attention on the most significant stimulation at the expense of less important stimuli [14]. Thus, it may be that stronger stimulation of the same target tissues might produce the increase in auditory thresholds which we had predicted. However, this hypothesis remains to be tested.

A limitation of this study is that while blinding of subjects receiving sham TENS appeared to be effective, blinding of subjects receiving authentic TENS was completely ineffective. This is, of course, the bane of studies using sensory stimulation as an intervention. However, the lack of blinding was apparently, and literally, inconsequential as the intervention produced no effect.

## Conclusions

Innocuous somatic stimulation in the form of TENS applied to the T4/T5 paraspinal muscles does not modulate hearing acuity in healthy young adults.
